# Reversible Gynecomastia and Hypogonadism Due to Usage of Commercial Performance-Enhancing Supplement Use

**DOI:** 10.1210/jcemcr/luae148

**Published:** 2024-08-14

**Authors:** Serena Chong, Catherine A Woolnough, Sundar R Koyyalamudi, Nimalie J Perera

**Affiliations:** Department of Endocrinology, Royal Prince Alfred Hospital, Sydney, New South Wales 2050, Australia; Department of Chemical Pathology, NSW Health Pathology, Royal Prince Alfred Hospital, Sydney, New South Wales 2050, Australia; Institute of Endocrinology and Diabetes, The Children's Hospital, Westmead, New South Wales 2145, Australia; Department of Endocrinology, Royal Prince Alfred Hospital, Sydney, New South Wales 2050, Australia; Department of Chemical Pathology, NSW Health Pathology, Royal Prince Alfred Hospital, Sydney, New South Wales 2050, Australia; Sydney Medical School and Charles Perkins Centre, University of Sydney, Sydney, New South Wales 2050, Australia

**Keywords:** gynecomastia, hypogonadism, performance enhancing supplements

## Abstract

Commercially available performance-enhancing supplements can contain banned performance-enhancing drugs (PEDs) and undisclosed steroid hormones that can induce hormonal abnormalities with associated clinical signs. We present a case of a 40-year-old male who developed bilateral gynecomastia and biochemical hypogonadotropic hypogonadism with a corresponding 6-month history of consuming commercially available performance-enhancing supplements for gym workouts. These performance-enhancing supplements were found to contain amounts of RAD-140, a selective androgen receptor modulator, MK-677, a GH secretagogue and cardarine, all of which are banned PEDs. In vitro analysis also detected undisclosed hormones testosterone, estradiol, and GH in all 3 supplements, with further steroid analysis using liquid chromatography mass spectrometry identifying an unidentified compound coeluting close to the testosterone peak. Cessation of these supplements led to full resolution of symptoms including normalization of hypogonadotropic hypogonadism. This case highlights the need for clinicians to consider commercially available performance-enhancing supplements as potential sources of PEDs and exogenous steroid hormones that can have adverse clinical consequences.

## Introduction

Performance-enhancing drugs (PEDs) are used to enhance the appearance of an individual and improve their physical capabilities such as endurance. The most recognized group of PEDs are nonprescribed anabolic-androgenic steroids (AAS) such as testosterone (1). More recently, selective androgen receptor modulators (SARMs), another type of PED, have gained popularity as an alternative to anabolic-androgenic steroids for muscle and strength development. SARMs are small synthetic ligands that bind to androgen receptors and, depending on their structure, function either as agonists, partial agonists, or antagonists. Examples include ostarine (MK-2866), ligandrol (LGD-4033), and testolone (RAD-140) (2). SARMs are perceived to be a safer alternative to AAS as they have fewer androgenic effects due to the substances not being metabolized to dihydrotestosterone by 5-alpha reductase or to estrogen by aromatase (3). There are limited clinical studies evaluating the pharmacokinetics profile, adverse effects, and drug interactions of SARMs. Apart from anecdotal evidence, the long-term effects remain largely unknown.

SARMs are commonly abused in conjunction with other drugs such as GH secretagogue or diuretics (1). Despite being banned by the US Food and Drug Administration and World Anti-Doping Agency, these substances can be found within commercially available performance-enhancing supplements, with a growing use of such supplements in the fitness and body-building communities.

## Case Presentation

A 40-year-old biological male of Middle Eastern descent presented to the endocrine outpatient clinic for evaluation of bilateral gynecomastia confirmed ultrasonographically. He had a 6-month history of breast tenderness and enlargement with no nipple discharge, with no previous or family history of pituitary or testicular disorders. He did not have any medical conditions and underwent a normal puberty with no history of infertility; he had biological children. He was not on any regular medication, with no history of smoking, alcohol abuse, or illicit drug use. He did not report reduced libido, erectile dysfunction, loss of hair, mood changes, headaches, or visual field changes. On examination, he had a body mass index of 23Kg/m^2^. There was palpable firm glandular tissue around the nipple areolar complex bilaterally. There was no nipple inversion, and he had normal size testes. His liver edge was not palpable.

## Diagnostic Assessment

Initial blood tests demonstrated low-normal gonadotrophins with low total testosterone calculated free testosterone levels and SHBG levels. A pituitary panel including prolactin 6.53 µg/L (ng/mL) [reference range (RR) 3.99-23.5 µg/L (ng/mL)], cortisol 154 nmol/L (5.58 µg/dL) [RR 70-650 nmol/L (2.54-23.56ug/dL)], adrenocorticotropic hormone 2.2 pmol/L (10 pg/mL) [RR < 12.10 pmol/L (<55 pg/mL)], IGF-1 34 nmol/L (260 ng/mL) [RR 12-34 nmol/L (92-260 ng/mL)], GH 1.165 µg/L (1.165 ng/mL) [RR 0-6 µg/L (0-6 ng/mL)], TSH 0.96mIU/L (0.96 µIU/mL) [RR 0.4-3.5mIU/L (0.4-3.5 µIU/mL)], and free T4 10.40 pmol/L (0.80 ng/dL) [RR 9-19 pmol/L (0.7-1.5 ng/dL)] (Table 1) with serum electrolytes, creatinine, liver enzymes, and full blood count all within normal limits. Repeat testing 3 weeks later showed similar results (Table 1). A testicular ultrasound was performed and showed no evidence of testicular atrophy (right testicular volume 14 mL, left testicular volume 12 mL, RR 12.5-19 mL) with normal echotexture and vascularity.

**Table 1.** Biochemical results for our patient

Hormone tested	Early September 2023 (on supplements)	Late September 2023 (on supplements)	Early November 2023 (6 weeks postcessation)	Early February 2024 (4 months postcessation)	Normal range
FSH	1.6 IU/L (1.6 IU/L)	**1.3 IU/L (1.3 IU/L)**	2.8 IU/L (2.8 IU/L)	2.2 IU/L (2.2 IU/L)	1.5-12.4 IU/L (1.5-12.4 IU/L)
LH	**1.0 IU/L (1.0 IU/L)**	**0.6 IU/L** **(0.6 IU/L)**	3.1 IU/L (3.1 IU/L)	1.6 IU/L (1.6 IU/L)	1.7-8.6 IU/L (1.7-8.6 IU/L)
Testosterone	**3.7 nmol/L (107 ng/dL)**	**3.1 nmol/L (89 ng/dL)**	10.3 nmol/L (297 ng/dL)	11.6 nmol/L (334 ng/dL)	8.6-29.0 nmol/L (248-836 ng/dL)
Free Testosterone (Calculated)*^a^*	**115 pmol/L (33.2 pg/mL)**	**90 pmol/L (26.0 pg/mL)**	—	252 pmol/L (72.9 pg/mL)	170-670 pmol/L (49-193 pg/mL)
SHBG	**8 nmol/L (0.7 µg/mL)**	**11 nmol/L (1.1 µg/mL)**	19.5 nmol/L (1.9 µg/mL)	27 nmol/L (2.6 µg/mL)	18.3-54.4 nmol/L (1.7-5.2 µg/mL)
Estradiol	—	—	**89 pmol/L (24.2 pg/mL)**	<80 pmol/L (<21.8 pg/mL)	<80 pmol/L (<21.8 pg/mL)

Abnormal values are shown in bold font. Values in parentheses are conventional units.

^*a*^Free testosterone (calculated) = 24.00314 × (Testosterone/Log_10_ SHBG) − 0.04599 × (Testosterone)^2^, using empirical formula from Sartorius et al (4).

On further questioning, the patient disclosed using 3 performance-enhancing supplements daily for his gym workouts, labeled B-140, B-677, and B-GW, which he purchased online and had used over the past 6 months, correlating with the onset of his breast symptoms. No other substances were disclosed. The patient was unaware of the active compounds in these supplements.

An extensive online search on these supplements revealed B-140, B-677, and B-GW to contain the active compounds RAD 140 (SARM), MK-677 (GH secretagogue), and cardarine, respectively. These supplements were sent for in vitro analysis; using methods described in (5), 10 mg/mL of supplement powder was mixed with PBS for 10 minutes and then centrifuged. The supernatant was tested on Roche Cobas, IMMULITE, and Liaison immunoassay platforms for the following analytes: cortisol, dehydroepiandrosterone sulfate, estradiol, testosterone (all with Roche Cobas), GH with Immulite2000, and IGF-1 with Liaison XL. Testosterone, GH, and estradiol were detected in all 3 supplements (Table 2). In particular, B-GW contained a substance that cross-reacted significantly with the estradiol assay. Further analysis to screen the powder in each of the capsules using Xevo TQ-XS liquid chromatography-tandem mass spectrometry following addition of internal standards and extractions using a hexane:ethyl acetate mixture (80:20 V/V) and acetonitrile:methanol (50:50 V/V) and reconstituted with methanol for steroid analysis and extraction using methyl tertiary-butyl ether in place of hexane:ethyl acetate mixture (80:20 V/V) for estradiol analysis was performed with an unidentified compound coeluting close to the testosterone peak noted. This unidentified compound was thus suspected to have cross-reacted with the laboratory immunoassay.

**Table 2.** In vitro results following 10 mg/mL of supplement mixed in buffer measured using laboratory immunoassays

Supplement name	Cortisol	DHEAS	Estradiol	Testosterone	GH	IGF-1
B-140	<1.50 nmol/L (<0.05 µg/dL)	<0.01 µmol/L (<0.19 µg/dL)	42 pmol/L (11.44 pg/mL)	0.43 nmol/L (12.50 ng/dL)	0.35 µg/L (0.11 ng/mL)	<0.39 nmol/L (<2.98 ng/mL)
B-677	<1.50 nmol/L (<0.05 µg/dL)	<0.01 µmol/L (<0.19 µg/dL)	128.00 pmol/L (34.90 pg/mL)	0.36 nmol/L (10.40 ng/dL)	0.37 µg/L (0.12 ng/mL)	<0.39 nmol/L (<2.98 ng/mL)
B-GW	<1.50 nmol/L (<0.05 µg/dL)	<0.01 µmol/L (<0.19 µg/dL)	305.00 pmol/L (83.08 pg/mL)	0.56 nmol/L (16.25 ng/dL)	0.34 µg/L (0.02 ng/mL)	<0.39 nmol/L (<2.98 ng/mL)

Conventional units are reported in parentheses.

Abbreviation: DHEAS, dehydroepiandrosterone.

## Treatment

The patient was advised to cease taking these supplements and have a progress review with repeat blood tests in 6 weeks. This demonstrated improvement in the gonadotrophin and testosterone levels with an LH of 3.1 IU/L (3.1mIU/L) [RR 1.7-8.6 IU/L (1.7-8.6mIU/L)], FSH of 2.8 IU/L [RR 1.5-12.4 IU/L (1.5-12.4mIU/L)], total testosterone improved to 10.3 nmol/L (297 ng/dL) [RR 8.6-29.0 nmol/L (248-836 ng/dL)], SHBG 19.5 nmol/L (1.85 µg/mL) [RR 18.3-54.4 nmol/L (1.73-5.18 µg/mL), and estradiol 89 pmol/L (24.24 pg/mL) [RR <80 pmol/L (<22 pg/mL)]. It was also noted that he had clinical improvement in his breast discomfort.

## Outcome and Follow-up

Four months after stopping the supplements, the patient reported complete resolution of his symptoms. Blood tests showed normal levels of hormones tested in the aforementioned pituitary panel, normal gonadotrophin and testosterone levels, and an undetectable estradiol level. The patient remained asymptomatic with no clinical gynecomastia at a subsequent review another 4 months later.

## Discussion

Increased recreational use of PEDs such as SARMs and GH secretagogues marketed as commercially available performance-enhancing supplements, coupled with a limited understanding of their adverse effects, has been a growing public health concern over the past decade. Although PED usage has historically been associated with elite athletes, most of them are now used by nonathletes in the form of these supplements (1). In the United States and Australia, such supplements have not been approved for recreational use (6). Despite this, many of these performance-enhancing supplements remain readily available for purchase online (2). These supplements commonly contain undisclosed and unapproved substances, increasing the risk of undesirable adverse effects (7).

The first supplement our patient consumed contained testolone (RAD-140). RAD-140 is one of several SARMs with a growing popularity among recreational users as an anabolic steroid to promote muscle growth. RAD-140 is currently undergoing testing as a potential treatment for androgen receptor positive metastatic breast cancer (8, 9) through suppression of estrogen receptor 1 and its downstream pathway (9). Due to fewer observed effects such as gynecomastia or testicular atrophy, SARMs are often mistaken by recreational users as a safer alternative compared to AAS (3). In the literature, RAD-140 has been associated with liver toxicity (10) and myocarditis (11). Other SARMs have been linked to a potential increased risk of myocardial infarctions and strokes (12). Our patient did not experience these adverse effects, but this does not preclude such a possibility in other cases. The second supplement contained Ibutamoren (MK-677) a potent GH secretagogue. Recreationally, MK-677 is used in addition to AAS and SARMs as a muscle-bulking agent. Similar to RAD-140, the long-term safety data of MK-677 remains unknown but has been associated with congestive heart failure (13). The third supplement contained cardarine, a peroxisome proliferator-activated receptor agonist. Recreationally, cardarine has been used to enhance fat loss in conjunction with an anabolic agent. While there is very limited immediate and long-term health data, cardarine has been associated with colorectal cancer (14). RAD-140, MK-677, and cardarine have not been approved for recreational use in the United States or in Australia by the Food and Drug Administration (15, 16) and the Therapeutic Goods Administration, respectively (17-19).

Consumers may not be aware that these “supplements” contain not only banned PEDs but also nondisclosed hormones. In vitro analysis of the 3 supplements in this case revealed them to also contain substances that cross-reacted with the immunoassays for estradiol, testosterone, and GH. None of the 3 supplements listed any of these substances as an active compound. Long-term testosterone abuse may cause irreversible damage to testicular function (20). Estradiol may lead to gynecomastia, testicular atrophy, and poor libido in men (21). The systemic side effects of GH abuse can result in hypertension, diabetes, and peripheral neuropathy, and features similar to acromegaly with gynecomastia have been reported (22). As the patient recovered upon cessation of these supplements, we believe the undisclosed estradiol, testosterone, GH, and MK-677 could have led to our patient's presentation of gynecomastia and biochemical hypogonadism.

Limitations of our case study include not having a measured serum estradiol level while on the supplements. Due to the potential partial solubility of these supplements in our in vitro study, the true levels of these hormone levels were likely to be much higher. Further testing of the supplements in a reputed nonbiological testing authority would be useful.

In conclusion, our case highlights the need for clinicians to consider seemingly harmless “supplements” as a source of banned PEDs and undisclosed exogenous steroid hormones. PEDs may have unknown adverse effects and may carry significant health risks. In addition, steroid hormones may be present at levels sufficient to induce clinical symptoms, especially in males. It is therefore important that clinicians routinely screen performance-enhancing supplements and consider them as a potential source of drugs and hormonal excess. Early recognition and management are useful to prevent harms related to use of performance-enhancing supplements.

## Learning Points

- There is limited reliable data on the immediate and long-term health impacts of PEDs such as SARMs.
- Clinicians should be aware that commercially available performance-enhancing supplements can potentially contain banned PEDs and undisclosed exogenous steroid hormones.
- Early recognition and management are useful to prevent harms related to use of performance-enhancing supplements.

## Data Availability

Original data generated and analyzed during this study are included in this published article.
